# Relationship between recurrently elevated hsCRP and adverse cardiovascular events among depressed patients in China: a time-to-event analysis

**DOI:** 10.3389/fcvm.2025.1554897

**Published:** 2025-10-17

**Authors:** Ying Wu, Yuwei Mi, Hanbin Cui, Qifa Song, Liemin Ruan

**Affiliations:** ^1^ Department of Psychosomatic Medicine, The First Affiliated Hospital of Ningbo University, Ningbo, Zhejiang, China; ^2^ Key Laboratory of Precision Medicine for Atherosclerotic Diseases, The First Affiliated Hospital of Ningbo University, Ningbo, Zhejiang, China; ^3^ Medical Data Center, The First Affiliated Hospital of Ningbo University, Ningbo, Zhejiang, China

**Keywords:** hsCRP, inflammation, depression, time-to-event analysis, adverse cardiovascular events

## Abstract

**Context:**

Persistent inflammation has been considered a biological link between depression and cardiovascular diseases(CVDs). Multipoint assessments of inflammation provide a more reasonable understanding of an individual's inflammatory status compared to single-point measurements. However, few studies have established strategies to investigate multipoint measurements of plasma high-sensitivity C-reactive protein(hsCRP).

**Aims/Objective/Hypothesis:**

To elucidate the association between recurrent elevations in hsCRP and cardiovascular events among depressed patients.

**Methods:**

This retrospective cohort study analyzed medical records over a ten-year follow-up to evaluate the association between longitudinal hsCRP patterns and recurrent cardiovascular events in patients with depression. An age-adjusted gamma frailty time-to-event model was used to assess the risk for three primary outcomes: chronic ischemic heart disease (CIHD), atrial fibrillation (AF) and other arrhythmias, and major adverse cardiac events (MACE). The cumulative incidence of these recurrent events was estimated using the Mean Cumulative Function (MCF).

**Results:**

The study included 10,770 patients [7,428 (68.97%) females]. Patients were classified into five groups based on hsCRP levels: hsCRP < 3 mg/L (*n* = 4,209, 39.08%), 3–8 mg/L (*n* = 1,697, 15.76%), one measurement of hsCRP ≥ 8 mg/L (*n* = 3,007, 27.92%), two to three measurements of hsCRP ≥ 8 mg/L (*n* = 1,349, 12.53%), and >3 measurements of hsCRP ≥ 8 mg/L (*n* = 508, 4.72%). The MCFs for CIHD across the five groups were 1.156, 1.339, 1.417, 2.021, and 2.36, respectively. For AF and other arrhythmias, the corresponding MCFs were 0.796, 1.369, 1.008, 0.858, and 1.578, while for MACE, they were 0.084, 0.089, 0.134, 0.196, and 0.172. Compared with the reference group (hsCRP < 3 mg/L), the adjusted hazard ratios (HRs) for CIHD were 1.28 (*P* = 0.14), 1.19 (*P* = 0.17), 1.70 (*P* < 0.001), and 1.88 (*P* < 0.001) across the other four groups; for AF and other arrhythmias, they were 1.38 (*P* = 0.07), 1.00 (*P* = 0.99), 1.04 (*P* = 0.84), and 1.83 (*P* < 0.01); and for MACE, they were 0.85 (*P* = 0.65), 0.73 (*P* = 0.24), 1.21 (*P* = 0.49), and 1.28 (*P* = 0.40), respectively.

**Conclusions:**

The gamma frailty time-to-event model indicated a link between persistent inflammation and cardiac events. Recurrent hsCRP elevations were more strongly associated with cardiovascular events than those observed in cross-sectional analyses.

**Trial Registration:**

NCT06239246; ChiCTR2400089334.

## Introduction

Depression and cardiovascular diseases (CVDs) frequently exist as comorbidities in a large proportion of patients. Depression has been identified as a risk factor for incident coronary heart disease (CHD), and the hazard ratios (HRs) or relative risks ranged from 1.6–1.8 (1–3). Multiple studies have reported an increased incidence of major depression in patients with established CHD and other CVDs (4, 5). A structured clinical interview revealed that 19.8% (95% CI, 19.1%–20.6%) of patients who recently experienced acute myocardial infarction (AMI) have major depression (4).

Systemic chronic inflammation, particularly low-grade inflammation, has been identified as a biological link between depression and CVD. Compared with non-depressed individuals, patients with depression show elevated levels of proinflammatory cytokines, including interleukin-1(IL1), interleukin-6(IL6), and tumor necrosis factor-alpha(TNF-α) (6, 7), as well as the systemic inflammation biomarker C-reactive protein(CRP) (8). High-sensitivity CRP (hsCRP) is commonly measured in patients with CVD and depression. A threshold of 3.0 mg/L for hsCRP is adopted to identify low-grade inflammation (9) following the recommendations of the American Heart Association (AHA).

Plasma CRP is a product of an immune response that promotes phagocytosis by macrophages, clearing necrotic and apoptotic cells and bacteria (10). Elevated CRP concentrations are common in individuals with various pathophysiological anomalies. Given that persistently elevated hsCRP levels are markers of chronic inflammation or underlying health issues (11, 12), numerous studies have used single measurements of hsCRP levels as the basis for stratification. However, the cross-sectional single-point measurement of CRP is not an appropriate index to evaluate the continuous impact of elevated CRP on depression and CVD. Hence, the establishment of a multipoint measurement analysis strategy for hsCRP requires further investigation.

In this study, we conducted a retrospective observational study involving patients who were first diagnosed with depression at a municipal hospital in China to investigate the associations between recurrently elevated hsCRP and various CVDs. Through time-to-event analysis, we aim to demonstrate the advantages of multipoint over single-point hsCRP measurements in reflecting the relationship between persistent inflammation and diseases.

## Methods

### Study design

This study investigated the associations between recurrently elevated hsCRP levels (defined as >3 mg/L) and CVD events using a frailty model of time-to-event analysis in patients with depression (13). The total follow-up duration was divided into 30-day intervals. Patients may experience multiple CVD events, which distinguishes this study from traditional survival analyses that focus on a single endpoint. As an observational study conducted in a real-world setting, this research often encounters interval and right censoring among patients. A gamma frailty model is appropriate for analyzing this type of data involving recurrent events, assuming that these events are independent of covariates. In this study, patient visits for CVDs were used as primary outcomes and estimated using the mean cumulative function (MCF) calculated by the Nelson-Aalen estimator of the cumulative hazard rate function (14). The MCF is a time-to-event analysis estimate that provides insights into the continual impact of chronic inflammation on the risk of adverse cardiovascular events in patients with depression (15). A classification based on hsCRP levels and the frequency of its elevation was used as a risk variable in the gamma frailty model to capture persistent inflammation. This allowed for the comparison of the incidence of various CVDs among the groups. Additionally, we performed a cross-sectional analysis of CVD occurrences at baseline using the same classification.

### Data source

The current study utilized the medical records of patients initially diagnosed with depression at the Psychosomatic Department of the First Affiliated Hospital of Ningbo University from 2015–2025. Patients were identified by searching for the term “depression” in their diagnoses, which included major depressive disorder (MDD), depressive state, depressive episode, mild depression, and moderate depression. The inclusion criteria were as follows: (1) a recorded clinical diagnosis of a psychological disorder involving depression, which was established by clinicians via structured interviews with the Patient Health Questionnaire-9 (PHQ-9) score ≥10 serving as a key diagnostic criterion; (2) age between 30 and 75 years; (3) at least one hsCRP record. Conversely, the exclusion criteria were as follows: severe psychiatric comorbidities such as bipolar disorder or schizophrenia; current engagement in psychotherapy; severe or life-threatening chronic conditions (e.g., end-stage renal disease, advanced liver disease, or active malignancy); cognitive impairment; pregnancy or lactation; or refusal to participate. Clinical features included demographic data, laboratory examinations, and diagnoses of depression and CVDs during follow-up in both outpatient and inpatient departments.

The data were de-identified, and their use was approved by the Ethics Committee of The First Affiliated Hospital of Ningbo University (Approval No. 2022-R-062). This study adhered to the Declaration of Helsinki.

### Primary outcomes

The primary outcome was a composite of adverse cardiovascular events, including chronic ischemic heart disease (CIHD), arrhythmias, and major adverse cardiac events (MACE). To identify these events, relevant diagnoses recorded on the hospital's database platform were standardized using their corresponding International Classification of Diseases, Tenth Revision (ICD-10) codes. For the purposes of this analysis, all diagnoses of CHD were classified under CIHD (ICD-10 code: I25); arrhythmias were defined as atrial fibrillation (AF) and other arrhythmias (ICD-10 codes: I44-I49); and MACE was a composite of AMI (ICD-10 codes: I20-I24) and heart failure (ICD-10 code: I50).

The diagnoses of these CVDs were validated by qualified clinical professionals based on a review of medical records, including surgical angiography, echocardiograms, and electrocardiograms. Furthermore, quality control for data recording and verification was ensured through a two-person review process.

### Laboratory measurements

After an overnight fast, plasma samples were collected. Glucose, total cholesterol (TC), high-density lipoprotein cholesterol (HDL-C), and triglycerides (TG) were analyzed on an automated biochemical analyzer using standardized enzymatic colorimetric methods. All procedures conformed to the recommendations of the International Federation of Clinical Chemistry and Laboratory Medicine (IFCC). White blood cell (WBC) counts were determined with an automated hematology analyzer. HsCRP was measured using the turbidimetric scattering method and reported in mg/L.

### Stratification of hsCRP

For the analysis, the entire follow-up period, including the initial 30-day baseline interval, was divided into sequential 30-day windows. For each window, a single representative measurement was determined. If multiple hsCRP readings were available, they were averaged. If no readings were available, the measurement for that window was considered not elevated. Subsequently, patients were classified into one of five unified groups based on their hsCRP levels throughout the entire follow-up: the “<3 mg/L” group (all representative measurements remained <3.0 mg/L), serving as the reference; the “3–8 mg/L” group (no measurement ≥8.0 mg/L, with a maximum level between 3.0 and 8.0 mg/L); the “1 time (≥8 mg/L)” group (exactly one 30-day window with a measurement ≥8.0 mg/L); the “2–3 times (≥8 mg/L)” group (two or three 30-day windows with a measurement ≥8.0 mg/L); and the “>3 times (≥8 mg/L)” group (more than three 30-day windows with a measurement ≥8.0 mg/L).

### Statistical analysis

For the three primary outcomes, we visualized event rates using event plots and MCF plots, with patients stratified by the five unified hsCRP groups. HRs and 95% confidence intervals (95% CIs) for the risk of adverse cardiovascular events were calculated using a gamma frailty model (rateReg function in the reda package) (16). When assessing the risk for the hsCRP groups, the model adjusted for age in 10-year intervals. The distinct HRs for the key risk factors of male sex and the “60–75 years” age group are also presented separately in the figure. To validate the model, the analysis was repeated in a random subsample comprising 25% of the total cohort.

Continuous variables were described as means and standard deviations (SDs). When values were normally distributed, the independent group t-test was used to compare the means of continuous variables between groups; otherwise, the Mann–Whitney test was applied. Categorical variables were described as counts and percentages. The Fisher's exact test was used to compare the proportions of categorical variables. A two-sided α of less than 0.05(*P* < 0.05) was considered statistically significant. All data analyses were performed using R software (version 4.3).

## Results

### Description of the cohort

The present study included 10,770 patients (7,428 females, 68.97%) who were diagnosed with depression and had at least one hsCRP measurement (Table 1). The time of the first measurement was designated as the beginning time of the follow-up and the baseline time. Among 10,770 patients, there were 4,209 with hsCRP < 3 mg/L, 1,697 with 3–8 mg/L, 3,007 with 1 time (≥8 mg/L), 1,349 with 2–3 times (≥8 mg/L), and 508 with >3 times (≥8 mg/L), respectively.

**Table 1:** Baseline clinical features and incident diagnoses of the study cohort, categorized by hsCRP level.

hsCRP(mg/L) at baseline						
Characteristics	<3	3–8	1 time (≥8)	2–3 times (≥8)	>3 times (≥8)	*P*
No.	4,209	1,697	3,007	1,349	508	-
Female	2,966 (70.47%)	1,189 (70.06%)	2,047 (68.07%)	913 (67.68%)	313 (61.61%)	<0.001
Age(y)	46.8 (12.76)	46.65 (13.49)	47.1 (13.91)	49.12 (14.51)	53.34 (14.7)	<0.001
hsCRP(mg/L)	1.09 (0.68)	3.6 (2.17)	19.6 (28.99)	18.08 (29.71)	19.76 (37.34)	<0.001
WBC (×10^9^/L)	6.51 (2.27)	6.97 (2.64)	7.62 (5.55)	7.7 (3.87)	8.12 (9.89)	<0.001
Glucose(mmol/L)	5.61 (1.52)	5.72 (1.76)	5.82 (1.93)	5.88 (2.18)	6.19 (3.02)	<0.001
TC(mmol/L)	5.07 (1.13)	5.06 (1.08)	4.99 (1.15)	5.05 (1.19)	5.13 (1.22)	<0.05
HDL-C(mmol/L)	1.29 (0.32)	1.27 (0.33)	1.26 (0.34)	1.24 (0.35)	1.2 (0.32)	<0.001
LDL-C(mmol/L)	3.08 (0.85)	3.06 (0.8)	3.03 (0.85)	3.02 (0.85)	3.07 (0.86)	0.102
TG(mmol/L)	1.47 (1.04)	1.51 (1.23)	1.5 (1.15)	1.56 (1.15)	1.53 (1.22)	0.201
Attack rate during the first year (%)						
CIHD	127 (3.02%)	53 (3.12%)	89 (2.96%)	66 (4.89%)	44 (8.66%)	<0.001
AF and other arrhythmias	109 (2.59%)	42 (2.47%)	80 (2.66%)	34 (2.52%)	30 (5.91%)	<0.001
MACE	16 (0.38%)	1 (0.06%)	8 (0.27%)	4 (0.30%)	2 (0.39%)	<0.001
Age and sex-adjusted relative risk (RR, 95% CI)						
CIHD	Reference	1.4	1.19	1.68	2.26	<0.001
AF and other arrhythmias	Reference	1.41	1.1	0.95	1.84	<0.001
MACE	Too few events					

Data are presented as mean (standard deviation) for continuous variables and as *n* (%) for categorical variables. AF, atrial fibrillation; CI, confidence interval; CIHD, chronic ischemic heart disease; hsCRP, high-sensitivity C-reactive protein; HDL-C, high-density lipoprotein cholesterol; LDL-C, low-density lipoprotein cholesterol; MACE, major adverse cardiovascular events; RR, relative risk; TC, total cholesterol; TG, triglycerides; WBC, white blood cell. –: Not applicable.

### Cross-sectional analysis of clinical features according to hsCRP levels at baseline

The baseline characteristics according to hsCRP levels are summarized in Table 1. Glucose and WBC levels showed a clear increasing trend with rising hsCRP levels (*P* < 0.001). Conversely, HDL-C levels showed a decreasing trend as hsCRP levels increased (*P* < 0.001). Although the absolute values for TC differed slightly among the groups (*P* < 0.05), no evident trend was observed. Similarly, no significant inter-group differences were found for LDL-C (*P* = 0.102) and TG (*P* = 0.201). After adjusting for age and sex, and using the <3 mg/L group as the reference, the relative risks (RR) for CIHD in the subsequent groups were 1.4, 1.19, 1.68, and 2.26, respectively. For AF and other arrhythmias, the corresponding RRs were 1.41, 1.1, 0.95, and 1.84. There were too few events to calculate the relative risk for MACE.

### Survival analysis by time-to-event analysis

Over the follow-up period, a positive association was observed between hsCRP levels and the attack rates of adverse events. For instance, the attack rate of CIHD rose from 10.31% in the hsCRP < 3 mg/L group to 26.77% in the >3 times (≥8 mg/L) group, while the rate of AF and other arrhythmias increased from 7.06%–21.06% across the same groups. Notably, MACE exhibited the most pronounced relative increase, with its attack rate escalating from 1.52%–4.92%.

**Figure 1:**
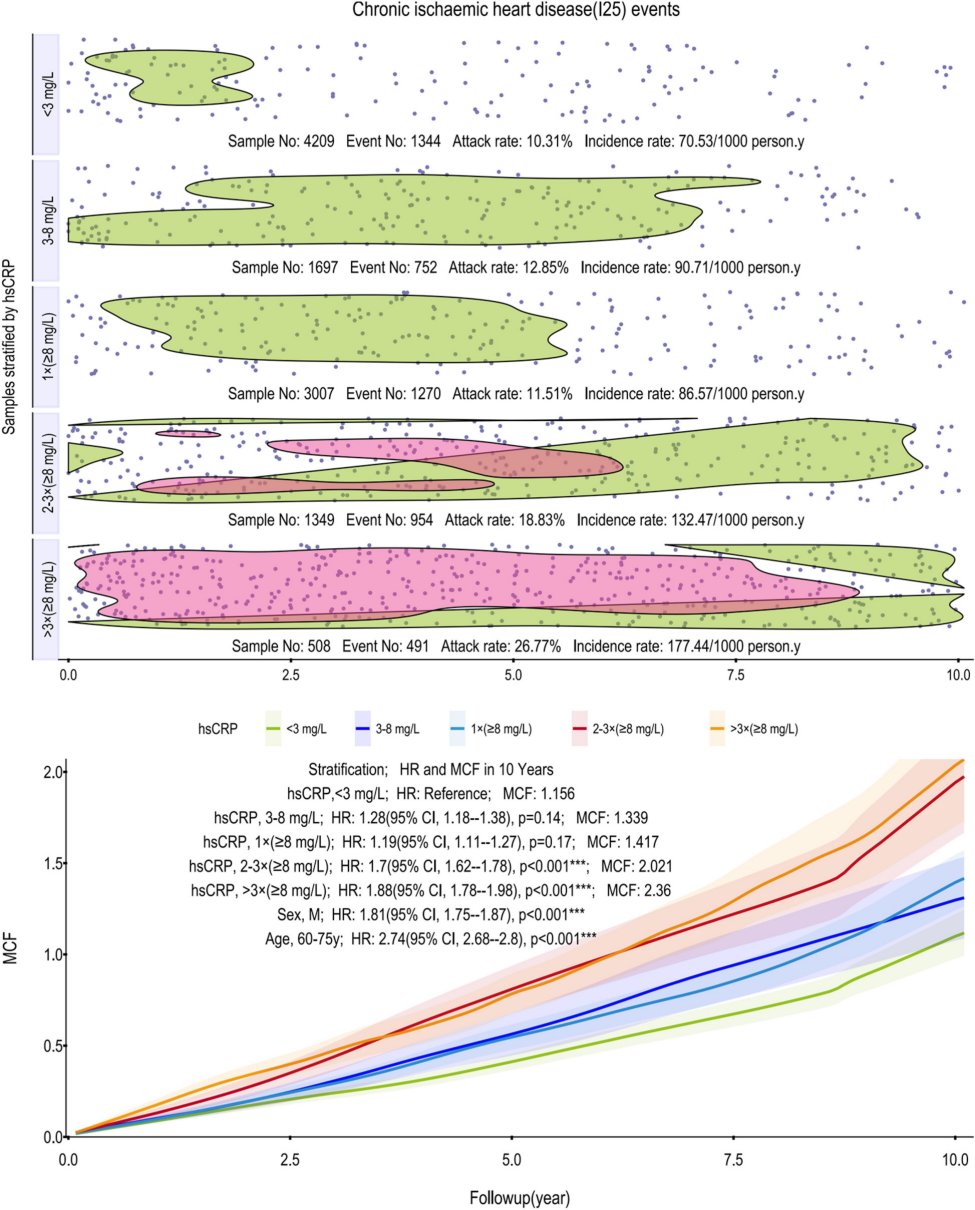
Scatter plot and Kaplan–Meier curve of the cumulative MCF estimates for CIHD. Cumulative incidence curves (MCF) and event distribution for CIHD, stratified by hsCRP levels over a 10-year follow-up. The upper panel shows the distribution of events and incidence rates for each hsCRP group. The lower panel displays the MCF curves. CIHD, chronic ischemic heart disease; hsCRP, high-sensitivity C-reactive protein; MCF, mean cumulative function; HR, hazard ratio; CI, confidence interval.

**Figure 2:**
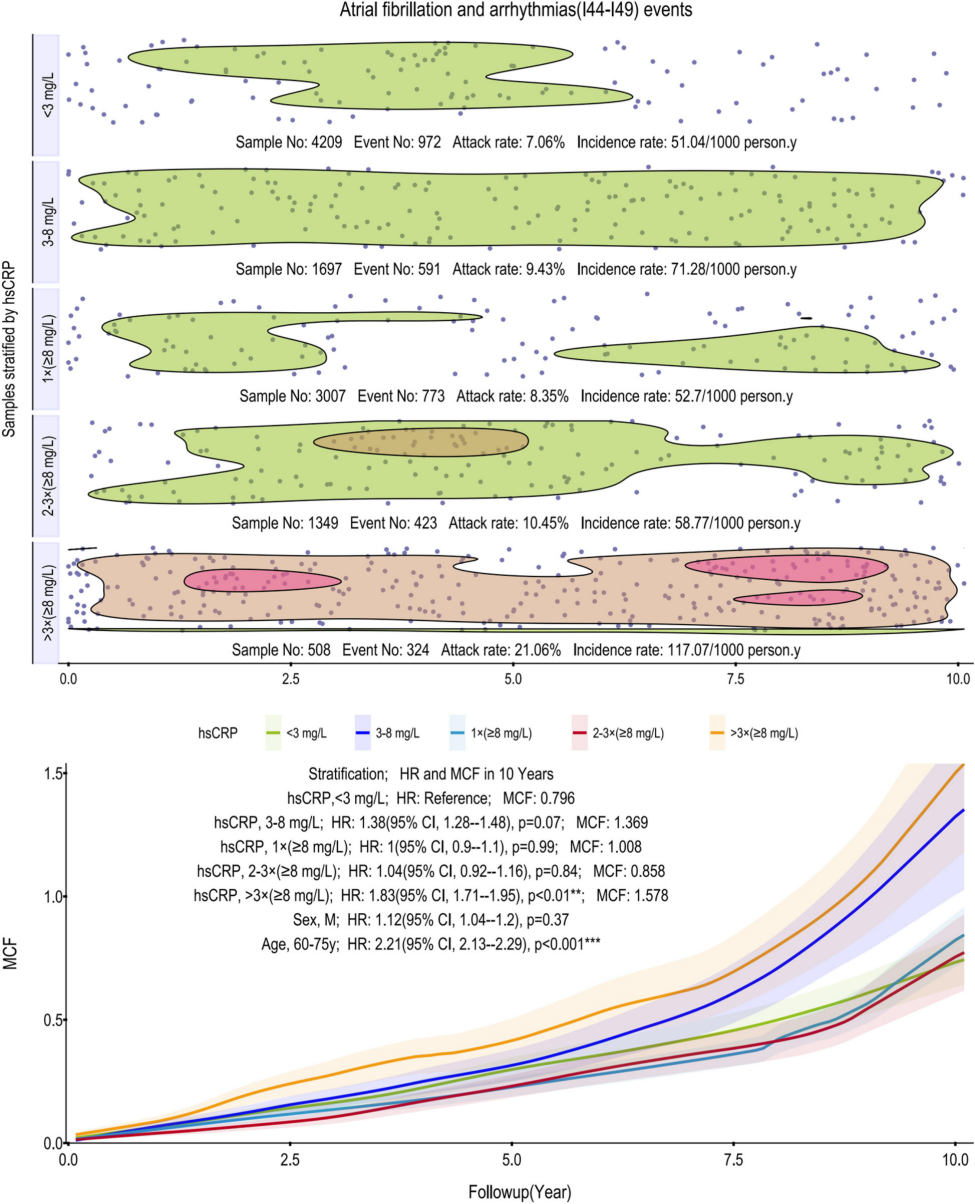
Scatter plot and Kaplan–Meier curve of the cumulative MCF estimates for AF and other arrhythmias. Cumulative incidence curves (MCF) and event distribution for AF and other arrhythmias, stratified by hsCRP levels over a 10-year follow-up. The upper panel shows the distribution of events and incidence rates for each hsCRP group. The lower panel displays the MCF curves. AF, atrial fibrillation; hsCRP, high-sensitivity C-reactive protein; MCF, mean cumulative function; HR, hazard ratio; CI, confidence interval.

**Figure 3:**
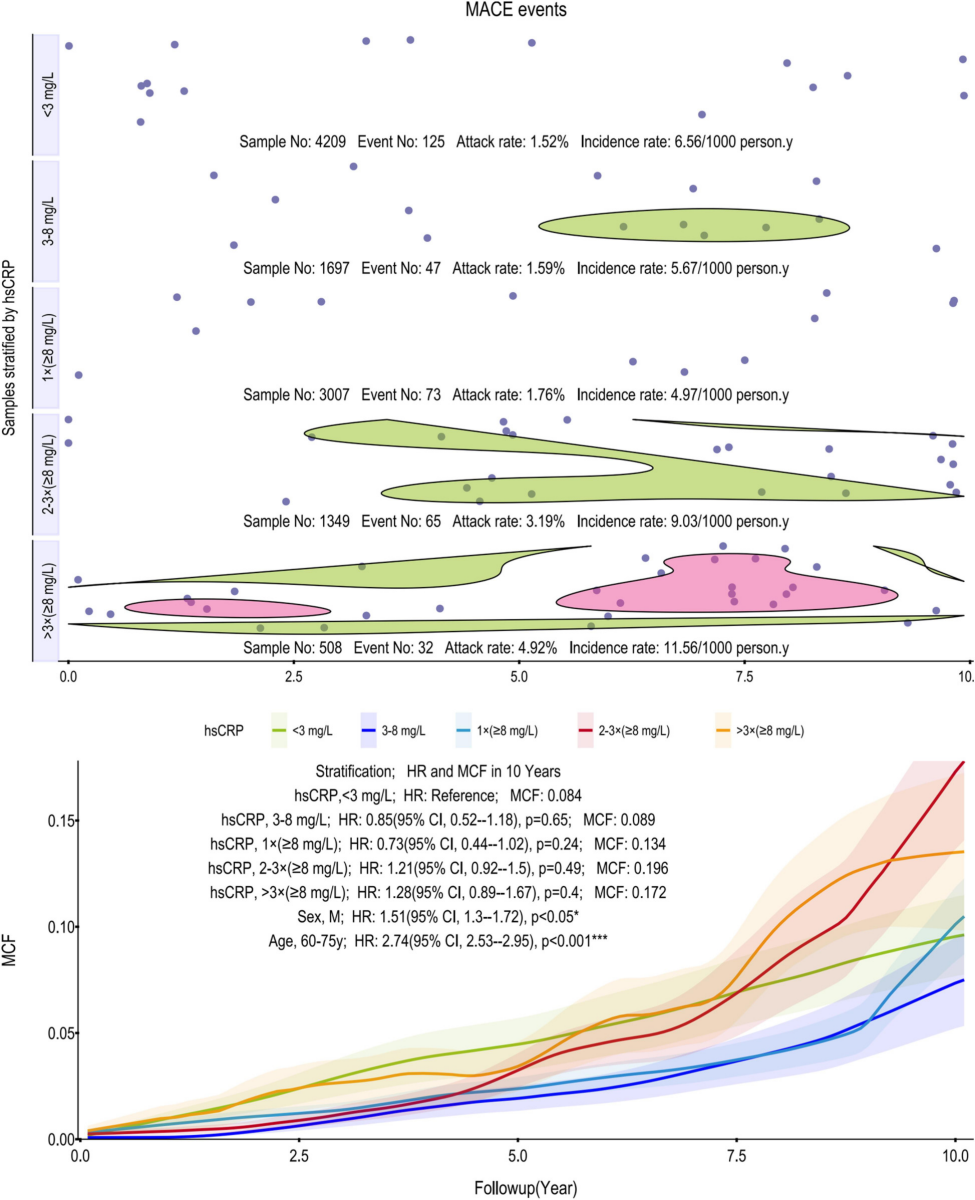
Scatter plot and Kaplan–Meier curve of the cumulative MCF estimates for MACE. Cumulative incidence curves (MCF) and event distribution for MACE, stratified by hsCRP levels over a 10-year follow-up. The upper panel shows the distribution of events and incidence rates for each hsCRP group. The lower panel displays the MCF curves. MACE, major adverse cardiovascular events; hsCRP, high-sensitivity C-reactive protein; MCF, mean cumulative function; HR, hazard ratio; CI, confidence interval.

To evaluate the cumulative incidence trajectories for CIHD, AF and other arrhythmias, and MACE, MCFs were calculated (Figures 1–3). All models were adjusted for age as a covariate, stratified in 10-year intervals.

As depicted in Figure 1, incidence rates of CIHD climb from 70.53–177.44 per 1,000 person-years across the hsCRP strata. Notably, the >3 times (≥8 mg/L) group exhibits a pronounced and sustained concentration of events, highlighted by a red-shaded region, which persists across the vast majority of the 10-year follow-up. The age-adjusted models underscored this risk, assigning the >3 times (≥8 mg/L) group a 10-year MCF of 2.36 and an HR of 1.88 (95% CI, 1.78–1.98; *p* < 0.001) relative to the reference group's MCF of 1.156 (<3 mg/L). Subgroup analyses further identified males (HR: 1.81, 95% CI, 1.75–1.87; *p* < 0.001) and older individuals (60–75 years; HR: 2.74, 95% CI, 2.68–2.80; *p* < 0.001) as particularly vulnerable.

For AF and arrhythmias, the relationship with hsCRP is notably non-linear. Incidence rates initially rise from 51.04–71.28 per 1,000 person-years, drop markedly, then surge to 117.07 in the >3 times (≥8 mg/L) group. This top stratum is characterized by two distinct high-density event clusters, one early and one late in the follow-up. This threshold effect was confirmed in age-adjusted models, where only the >3 times (≥8 mg/L) group reached statistical significance (HR: 1.83, 95% CI: 1.71–1.95; *p* < 0.01), with a 10-year MCF of 1.578. Among subgroups, age (60–75 years) remains a potent risk factor (HR: 2.21, 95% CI: 2.13–2.29, *p* < 0.001) (Figure 2).

Similarly, for MACE, the association with hsCRP was non-linear (Figure 3). Incidence rates initially decreased from 6.56 to 4.97 per 1,000 person-years before climbing sharply to 11.56 in the >3 times (≥8 mg/L) group. While the MCF curves showed a general separation, the >3 times (≥8 mg/L) group, which carried the highest point estimate of risk (HR = 1.28), did not translate into statistical significance in the age-adjusted models. None of the hsCRP strata reached a significant HR compared to the reference group. In contrast, subgroup analyses identified male sex (HR: 1.51, 95% CI, 1.30–1.72; *p* < 0.05) and older age (60–75 years; HR: 2.74, 95% CI, 2.53–2.95; *p* < 0.001) as potent and independent predictors of MACE.

To confirm these findings, we repeated the analyses for all three primary outcomes in a random subsample of one-quarter of the total population, and the conclusions remained unchanged.

## Discussion

Our study developed a methodological strategy to investigate the relationship between multipoint measurements of inflammation and adverse cardiac outcomes, highlighting the clinical significance of using multipoint hsCRP measurements to predict cardiovascular risk in patients with depression.

In medical research, subjects frequently experience multiple events, such as recurrent infections or hospitalizations. Frailty models effectively examine the dynamic incidence of these events, providing advantages over cross-sectional methods. The MCF, a statistical estimator used in survival analysis and related to frailty models, quantifies the average number of cumulative events occurring within a specified time frame (13). In analyzing recurrent cardiovascular events, MCF estimates effectively capture their dynamic nature, yielding more profound insights into patterns and trends (15, 17). Jung et al. demonstrated the efficacy of the joint frailty model in stratifying risk among HIV patients with advanced multidrug-resistant infections by accounting for unobserved heterogeneity. The study revealed that the high-risk group identified by this model exhibited an incidence rate approaching 70%, with a significant correlation between the recurrence of AIDS-defining events and mortality (18). Additionally, Han et al. employed the MCF method to analyze readmission patterns in patients with continuous-flow left ventricular assist devices, identifying the primary reasons for readmission over three years as bleeding (0.74 times/person), infection (0.7 times/person), device malfunction (0.52 times/person), arrhythmia (0.3 times/person), and right heart failure (0.28 times/person). Notably, the frequency of readmissions did not adversely affect survival rates [HR, 1.03 (95% CI, 0.92–1.17); *P* = 0.58], indicating that the MCF method effectively captures the complexity of recurrent event data (19). Therefore, this study aims to apply the MCF method to investigate the relationship between hsCRP and adverse cardiovascular events, potentially offering new insights into the field.

Our analysis shows that persistent, not transient, inflammation drives subsequent cardiovascular risk. A single elevated hsCRP reading had limited prognostic value, whereas two or more indicated a significantly higher risk for CIHD, AF and other arrhythmias, and MACE. Our findings highlight that for CIHD, persistent, high-grade inflammation is the critical predictor, whereas a single measurement is not. Specifically, while a single hsCRP reading ≥8 mg/L showed no significant association with future CIHD events (HR: 1.19, *p* = 0.17), a pattern of two or more such elevations robustly indicated a heightened risk (HR: 1.70 and 1.88, respectively; both *p* < 0.001) (Figure 1). This distinction is likely because the ≥8 mg/L threshold identifies a state of high-grade inflammation more directly involved in coronary atherosclerosis. Biologically, such an intense and persistent inflammatory state provides a stronger, more specific risk signal than lower-grade inflammation (3–8 mg/L, *p* = 0.14). Requiring multiple measurements confirms this signal's persistence over transient fluctuations, making it a more clinically meaningful strategy for CIHD risk stratification (20, 21). In stark contrast, this association was weaker for AF and other arrhythmias, where significant risk emerged only after more than three high measurements (HR 1.83, *p* < 0.01). For MACE, no significant association was observed at all, likely due to our cohort's low event rate and insufficient statistical power.

Our findings support and extend the substantial body of evidence linking inflammation to adverse cardiovascular outcomes. The inflammatory hypothesis of atherosclerosis was validated by the landmark CANTOS trial, which demonstrated that targeting the interleukin-1β pathway significantly reduced recurrent cardiovascular events, providing a clear rationale for inflammation as a therapeutic target (20). Furthermore, the significance of hsCRP is highlighted by its role as a mediator in the link between psychosocial factors and cardiovascular risk; meta-analyses have established that depression increases CHD risk, an association partly explained by low-grade inflammation indicated by elevated hsCRP (21, 22). This connection is particularly relevant to our work, as patients with CVD frequently exhibit comorbid depression and elevated hsCRP, especially among the elderly (23). This comorbidity is also linked to the dysregulation of immunometabolism (24, 25), which may contribute to adverse cardiovascular outcomes (26). Given that our inclusion criteria mandated that all patients had a first episode of depression, we could not perform a comparative analysis against a non-depressed group, an area that warrants further investigation. In this context, our results align with recent evidence demonstrating that elevated hsCRP is a risk factor for specific adverse outcomes, such as cardiac conduction disorders (27) and recurrent CHD events in patients with high inflammatory burden (28). Particularly noteworthy and resonant with our findings is the Jackson Heart Study. In that study, which focused on black adults, the authors reported that baseline hsCRP was not associated with incident heart failure (HR 1.08, 95% CI, 0.96–1.22). However, reflecting the importance of sustained inflammation, elevated hsCRP levels during repeated measurements were associated with an increased risk of overall heart failure (HR 1.22, 95% CI, 1.03–1.44) (29). Although this was a retrospective study, our research strengthens the causal inference between hsCRP and CVDs through the MCF method. The prespecified hsCRP data design, which focuses on recurrence, helps mitigate regression dilution bias (30).

Sex demonstrated a differential and endpoint-specific association with cardiovascular outcomes. For the CIHD endpoint, males exhibited a significantly higher risk (HR: 1.81, *p* < 0.001). Although considerable literature indicates that in specific contexts, such as depression, females may exhibit a stronger pro-inflammatory response (31), we propose that the powerful, lifelong cardioprotective effects of estrogen—through favorable modulation of lipids and endothelial function—provide a protective influence that predominates (32). Thus, the greater net atherogenic burden in males remains the principal driver of their increased risk. In contrast, for the composite arrhythmia endpoint, sex was not a significant predictor (HR: 1.12, *p* = 0.37). This may be explained by the interplay of competing, sex-specific risk factors; for instance, the male predisposition for AF (partly due to larger atrial diameters) (33, 34) may be balanced by a female predisposition for other arrhythmias associated with distinct electrophysiological properties, such as a longer QTc interval (35). For a broad composite endpoint, the confluence of these different risk profiles could lead to a non-significant association at the population level.

The primary limitation of this study is its reliance on real-world clinical diagnoses. On one hand, diagnostic application may have inherent variability across physicians, sites, and time. On the other hand, we lacked quantitative severity data; although an initial diagnosis required a PHQ-9 score of ≥10, the specific scores were not available for our analysis, thus precluding stratification by depression severity. Furthermore, as a single-center study with a female predominance, our findings have limited generalizability and require validation in larger, multi-center, and more diverse cohorts. Secondly, we did not examine other inflammatory markers, such as IL-6 and TNF-α. These markers have consistently shown elevated levels in meta-analyses of depressed populations and are among the most prominent elevations observed in circulating inflammatory markers (6, 36). Thirdly, data on prescribed medications, particularly for the treatment of depression, were not systematically collected or analyzed. This represents a critical limitation as many psychotropic medications, including certain antidepressants, are known to possess immunomodulatory or anti-inflammatory effects that can influence hsCRP levels (37–39). Consequently, pharmacotherapy acts as a potential unmeasured confounder in the relationship we observed between hsCRP and adverse cardiovascular outcomes. However, the large sample size, extended follow-up period, and novel longitudinal data analysis methods provide a strong foundation for the evidence linking recurrently elevated hsCRP levels to adverse cardiovascular outcomes.

In summary, longitudinal data from a municipal hospital in China indicate that, over a 10-year follow-up period, recurrent elevations in hsCRP are associated with an increased risk of adverse cardiovascular events, including CIHD, AF and other arrhythmias, and MACE, an association that exhibits varying degrees of impact. By moving beyond single-point hsCRP measurements, our findings offer a more dynamic view, capturing the cumulative risk posed by persistent inflammatory states. This underscores the importance of investigating such states, particularly in the context of comorbid CVD and mental disorders. We advocate that healthcare professionals effectively identify depressed patients with intermittently elevated inflammation levels and provide targeted treatment, as these patients are at an increased cardiovascular risk. For clinical application, a risk-stratified monitoring framework could be considered: this might involve an initial hsCRP screening for all patients, with low-risk individuals undergoing annual re-testing, while those with high initial levels could receive a follow-up test within 1–3 months to confirm persistency and guide more intensive intervention.

## Data Availability

The data analyzed in this study is subject to the following licenses/restrictions: The original data supporting this study can be accessed through the electronic medical record system of our hospital. The dataset is not suitable for inclusion in a publicly accessible repository due to concerns about patient privacy. Requests to access these datasets should be directed to the corresponding author(s).
